# 快速现场细胞学评价在EBUS-TBNA取样诊断肺癌中的价值

**DOI:** 10.3779/j.issn.1009-3419.2018.11.05

**Published:** 2018-11-20

**Authors:** 青 向, 涛 万, 前方 胡, 虹 陈, 岱容 李

**Affiliations:** 400016 重庆，重庆医科大学附属第一医院呼吸与危重症学科 Department of Respiratory and Critical Care Medicine, the First Affiliated Hospital of Chongqing Medical University, Chongqing 400016, China

**Keywords:** 超声支气管镜引导下的经支气管针吸活检术, 快速现场细胞学评价, 肺肿瘤, Endobronchial ultrasound-guided transbronchial needle aspiration, Cytological rapid on-site evaluation, Lung neoplasms

## Abstract

**背景与目的:**

在肺及纵隔占位性病变的患者中大部分临床考虑诊断为原发性肺癌，超声支气管镜引导下的经支气管针吸活检术(endobronchial ultrasound-guided transbronchial needle aspiration, EBUS-TBNA)是一种常用的获取病理组织明确诊断的手术。在EBUS-TBNA过程中，快速现场细胞学评价(cytological rapid on-site evaluation, C-ROSE)是一项有用的辅助技术。本研究探讨C-ROSE在EBUS-TBNA取样诊断肺癌中的价值。

**方法:**

对141例经胸部计算机断层扫描(computed tomography, CT)发现存在纵隔和(或)肺门病灶(包括肿大的淋巴结/肿块)且临床高度疑诊原发性肺癌而行EBUS-TBNA患者进行回顾性分析，其中术中行C-ROSE者81例，未行C-ROSE者60例，分析两组患者的穿刺情况及并发症发生率，同时分析C-ROSE联合EBUS-TBNA取样对肺癌诊断的敏感度、特异度、阳性预测值、阴性预测值。

**结果:**

C-ROSE组与非C-ROSE组穿刺针数及穿刺部位数无统计学差异，穿刺合格率分别为98.77%和90.00%(*P* < 0.05)，诊断率分别为88.89%和75.00%(*P* < 0.05)，并发症发生率分别为1.23%和11.67%(*P* < 0.05)。C-ROSE联合EBUS-TBNA对肺癌诊断的敏感度为92.21%，特异度为100.00%，阳性预测值为100.00%，阴性预测值为40.00%。

**结论:**

C-ROSE在疑诊肺癌行EBUS-TBNA中应用可以提高穿刺成功率及诊断率、减少并发症，值得推广。

## Abstract (en)

**Background and objective:**

Most of the patients with lung and (or) mediastinal occupying lesions are considered to be primary lung cancer clinically, and endobronchial ultrasound-guided transbronchial needle aspiration (EBUS-TBNA) is a commonly useful operation to obtain the tissue sample and get definitive diagnosis of pathological tissues. In the EBUS-TBNA process, cytological rapid on-site evaluation (C-ROSE) is a useful technology. The purpose of our study is to discuss the value of C-ROSE in the diagnosis of lung cancer by EBUS-TBNA sampling.

**Methods:**

Retrospective analysis of 141 cases clinical data who were performed with EBUS-TBNA and suspected diagnosis primary lung cancer, which were found have mediastinal and (or) lung lesions (including the enlargement of the lymph nodes/mass) by computed tomography (CT). Among these patients, 81 patients were in the C-ROSE group and 60 patients were in the No C-ROSE group. The message of puncture and complication of EBUS-TBNA with or without C-ROSE were compared. At the same time, we analysis the sensitivity and specificity, positive predictive value, negative predictive value of C-ROSE combined with EBUS-TBNA in that of the diagnosis of lung cancer.

**Results:**

We found no statistical difference of the needle passes between C-ROSE group and No C-ROSE group. But in C-ROSE group, specimen qualified rate and diagnostic yields were signicantly higher than No C-ROSE group (98.77% *vs* 90.00%, 88.89% *vs* 75.00%, *P* < 0.05), the incidence of complications in the C-ROSE group was signicantly lower than that in the No C-ROSE group (1.23% *vs* 11.67%, *P* < 0.05). The sensitivity, specificity, positive predictive value and negative predictive value of C-ROSE combined with EBUS-TBNA in the diagnosis of lung cancer are 92.21%, 100.00%, 100.00% and 40.00%.

**Conclusion:**

EBUS-TBNA combined with C-ROSE can improve the specimen qualified rate and diagnostic rate, also can reduce the complications thus worthy of further promotion.

在临床工作中经常遇到影像学提示存在纵隔和(或)肺门病灶(包括肿大的淋巴结/肿物)的患者，有经验的临床医师可以综合患者性别、年龄、吸烟史、肿瘤家族史、临床表现、体格检查、肿瘤标志物、影像学等资料分析后得出倾向性诊断，其中大部分临床考虑原发性肺恶性肿瘤可能性大，但须获取合适的组织标本明确病理诊断、指导进一步治疗。超声支气管镜引导下的经支气管针吸活检术(endobronchial ultrasound-guided transbronchial needle aspiration, EBUS-TBNA)对存在纵隔和(或)肺门病灶的诊断有较高价值，它包括穿刺肺门和/或纵隔淋巴结(肿大淋巴结EBUS-TBNA)和直接穿刺气管旁肿块(病灶EBUS-TBNA)。

快速现场评价(rapid on-site evaluation, ROSE)在当代呼吸介入学中是不可缺少的一部分，它具体是指在支气管镜检查等介入操作过程中由细胞病理学家现场对穿刺标本进行制片和染色并进行快速评价、向操作者反馈穿刺是否成功、提供初步诊断的一种方法^[1]^。ROSE技术包括快速现场细胞学评价(cytological ROSE, C-ROSE)和快速现场微生物学评价(microbiological ROSE, M-ROSE)，如今C-ROSE应用更为广泛。现国内外已经有许多文献报道了C-ROSE联合经支气管针吸活检术(transbronchial needle aspiration, TBNA)可以提高诊断率、减少无谓操作及并发症、节省费用^[2, 3]^。但是相较于操作时不能直接看到气管或支气管壁外结构类似“盲穿”的TBNA^[4]^，EBUS-TBNA将超声支气管镜与经支气管针吸活检术联合，让临床医生可以在超声实时监测下对病灶进行穿刺活检，穿刺过程中可以清楚地观察到病灶内的血流及其与周边血管的关系，避免误穿血管，使穿刺的准确性和安全性大大提高^[5]^。相较于TBNA，EBUS-TBNA会削弱C-ROSE在操作中的益处，那么在行EBUS-TBNA时联用C-ROSE是否可以获益呢？国内相关研究较少，国外研究结论不一^[6, 7]^，且缺乏专门针对肺癌的研究，本研究旨在探讨C-ROSE联合EBUS-TBNA针对疑诊肺癌患者的诊断价值。

## 1 资料与方法

### 1.1 临床资料

回顾性分析2013年11月-2017年11月于重庆医科大学附属第一医院气管镜室行EBUS-TBNA检查的所有患者。纳入标准：①术前行胸部计算机断层扫描(computed tomography, CT)显示纵隔/肺门病灶(包括短径 > 1 cm的淋巴结及肿块)；②综合患者病史及辅助检查等结果分析后高度疑诊原发性肺癌；③术前完善心电图、凝血功能等检查排除超声支气管镜检查相关禁忌证；④术前告知患者及患者家属手术操作过程及相关风险并签署手术同意书。排除标准：①综合患者病史及辅助检查结果分析后高度疑诊转移性肺癌、淋巴结结核、结节病、淋巴瘤者；②可通过常规支气管镜或体表肿大淋巴结穿刺活检等手段明确诊断者；③存在严重心、肺功能衰竭等手术禁忌者；④不能获取完整临床资料者。此次研究共纳入141例患者，其中行EBUS-TBNA联合C-ROSE者81例(C-ROSE组)，单独行EBUS-TBNA者60例(非C-ROSE组)，两组患者间一般资料无统计学差异(*P* > 0.05)(表 1)。

**1:** 两组患者的一般资料 General data for two groups of patients

	C-ROSE group (*n*=81)	No C-ROSE group (*n*=60)	*P*
Age (yr)	60.30±8.82	59.92±11.21	0.82
Gender (Male/Female)	59/22	45/15	0.77
History of smoking (Yes/No)	55/26	40/20	0.88
Ventilator (Yes/No)	14/67	10/50	0.92
ROSE: rapid on-site evaluation.			

### 1.2 操作过程

#### 1.2.1 EBUS-TBNA操作过程

术前由麻醉医师和手术医师综合患者年龄、基础疾病、肺功能、血气分析等情况决定术中麻醉方式：①轻度静脉镇静、镇痛+高流量鼻导管吸氧；②静脉镇静、镇痛后置入喉罩连接呼吸机辅助通气。检查前患者禁食禁饮4 h以上、开通静脉通道，术前利多卡因雾化吸入局部麻醉，术中追加芬太尼及咪达唑仑静脉镇静、镇痛，全程心电监护及指脉氧监测。术时先行常规支气管镜清理呼吸道，观察管腔情况，然后换用超声支气管镜，将凸面探头贴近气道壁达到目标位置，向水囊中注入适量的水，打开主机的超声模式并探查肿大的淋巴结和(或)肿物大小及部位，确定行淋巴结EBUS-TBNA和(或)病灶EBUS-TBNA，将超声支气管镜调整至合适的穿刺部位，测量病灶大小及穿刺距离，打开彩色多普勒超声模式并观察病灶内血供情况并确定其与邻近血管的关系，确定穿刺部位及穿刺方向，然后经过工作通道送入穿刺针(OLYMPUS NA-201SX-4021, 21 G)，每次穿刺连接负压空针反复抽吸20次左右。C-ROSE组根据快速现场细胞学判读结果、恶性细胞所占比例、临床有无特殊需求等实际情况确定穿刺针数，非C-ROSE组根据获得标本情况由手术医师凭经验确定穿刺针数。穿刺结束后将获得的所有组织条标本经10%甲醛固定送病理科行组织病理学检查(若穿刺未获得组织标本则不进行组织病理学检查)，并由病理科医师判断是否行免疫组化检查；将每次穿刺获得的细胞学涂片送细胞病理学检查。所有手术由我科气管镜室同一名有资质的手术医师操作。所有送检标本制片后分别由两名具有资质的病理科医师阅片后发布报告。

#### 1.2.2 C-ROSE操作过程

C-ROSE组的患者每次穿刺后由我科细胞病理室的细胞病理学医师现场制片：若穿刺后获得组织条样标本则连接负压空针将每次穿刺获得的组织条轻推至干净玻片中央，采用滚片法制片2张，1张用于C-ROSE，另1张固定后送细胞病理学检查；若仅穿刺抽吸获得细胞液标本，则连接负压空针，推出穿刺针内液体以喷片法制片2张，1张用于C-ROSE，另1张固定后送细胞病理学检查^[8]^。用于C-ROSE的玻片采用世界卫生组织(World Health Organization, WHO)推荐的标准ROSE染色方法——迪夫快速染色方法^[8, 9]^染色，由细胞病理学医师根据显微镜下直接涂片的细胞学特征行快速现场评估(20 s-40 s)，评估的三要素包括：①是否取到癌变细胞；②恶性细胞比例、癌变细胞数量是否足够；③细胞是否完整，并根据文献描述判读结果及满意度^[8]^。当C-ROSE判定穿刺标本为非诊断材料或恶性细胞比例低，或因临床需要获取更多靶标本行基因检测等情况时，则再次穿刺。若C-ROSE结果为阳性则在原部位穿刺，若C-ROSE结果为阴性则更换穿刺部位再次穿刺获取标本行C-ROSE分析，但每例患者总体穿刺针数不超过7针^[10]^。当现场阅片结果判定为穿刺到癌性标本且标本满意时，对满意标本进行恰当分检以确保获得明确的富含信息的诊断，记录C-ROSE诊断结果及镜下细胞学特征，结束操作。

### 1.3 诊断结果的判断

若EBUS-TBNA术后送检的穿刺组织标本或细胞学标本获得明确的病理诊断结果，则以此为最终诊断；若EBUS-TBNA术后病理诊断结果为阴性，但患者术后行经皮肺穿刺活检、胸腔镜、纵隔镜、开胸手术等有创操作获得明确的病理诊断，则以后者的病理结果为最终诊断；若EBUS-TBNA术后病例诊断结果为阴性，且患者因各种因素未能进一步行有创检查，根据临床诊断行经验性治疗，密切随访至少半年临床诊断得到验证后，以患者的临床诊断为最终诊断。

### 1.4 穿刺标本合格性判断

两组患者术后将获得的所有组织条及抽吸液送组织学及细胞学检查，患者的组织学或细胞学标本，若任一标本在镜下见到明显异型细胞、数个淋巴细胞团等则认为该患者穿刺标本合格；若患者的组织学及细胞学标本镜下均仅见到少量有核细胞，见大量血细胞、呼吸道粘膜细胞，则认为该患者穿刺标本不合格。

### 1.5 统计方法

以SPSS 22.0统计软件进行统计分析，计量资料的比较采用*t*检验，计数资料的比较采用卡方检验，以*P* < 0.05为有统计学差异。

## 2 结果

### 2.1 两组手术情况

C-ROSE组共81例患者，其中23例行病灶EBUS-TBNA(包括纵隔肿物3例次、右肺肿物15例次、左肺肿物5例次)，共穿刺55针，获得41条组织条(比例74.55%)；61例行肿大淋巴结EBUS-TBNA，共穿刺166针，获得129条组织条(比例77.71%)。非C-ROSE组共60例患者，其中有13例行病灶EBUS-TBNA(包括纵隔肿物3例次、右肺肿物8例次、左肺肿物2例次)，共穿刺32针，获得26条组织条(比例81.25%)；49例患者行肿大淋巴结EBUS-TBNA，共穿刺122针，获得91条组织条(比例74.59%)。两组患者镜下测量病灶或肿大淋巴结直径、平均穿刺部位数、平均穿刺针数、平均穿刺深度等结果详见表 2。具体穿刺淋巴结情况详见表 3。

**2:** 两组患者穿刺情况（Mean±SD） The message of two groups of patients puncture (Mean±SD)

Surgery^*^		C-ROSE group	No C-ROSE group	*P*
Tumor EBUS-TBNA	Tumor diameter (cm)	3.78±1.65	3.71±1.27	0.90
	Puncture numbers	2.39±0.99	2.46±1.05	0.84
	Puncture depth (cm)	2.27±0.45	2.21±0.33	0.52
Lymph node EBUS-TBNA	Lymph node short diameter (cm)	2.58±0.69	2.68±0.84	0.28
	Number of lymph nodes punctured	1.46±0.62	1.43±0.61	0.80
	Puncture numbers	2.72±1.16	2.49±1.02	0.28
	Puncture depth (cm)	2.03±0.27	1.97±0.40	0.14
^*^: Three patients had tumor EBUS-TBNA and lymph node EBUS-TBNA at the same time in C-ROSE group; two patients had tumor EBUS-TBNA and lymph node EBUS-TBNA at the same time in No C-ROSE group. EBUS-TBNA: endobronchial ultrasound-guided transbronchial needle aspiration.				

**3:** 两组患者穿刺淋巴结部位分布 The lymph nodes distribution of two groups of patients puncture

Lymph nodes	C-ROSE group (case-time)	No C-ROSE group (case-time)
2R	-	5(7.14%)
4L	6 (6.74%)	2 (2.86%)
4R	23 (25.84%)	22 (31.43%)
10L	2 (2.25%)	1 (1.43%)
10R	6 (6.74%)	6 (8.57%)
11L	12 (13.48%)	4 (5.71%)
11R	3 (3.37%)	1 (1.43%)
12R	-	1 (1.43%)
7	37 (41.57%)	28 (40.00%)
Total	89 (100.00%)	70 (100.00%)

### 2.2 最终诊断

C-ROSE组81例患者术后明确病理诊断者72例(包括病灶EBUS-TBNA明确诊断19例、淋巴结EBUS-TBNA明确诊断53例)，术后未明确诊断的9例患者中有5例进一步行有创操作后明确诊断(包括2例肺癌、1例淋巴瘤、2例炎性假瘤)，4例患者随访影像学提示短期病灶明显增大伴肺外新发病灶，结合典型的影像学表现临床诊断为肺癌；故C-ROSE组81例患者包括77例肺癌、2例淋巴瘤、2例炎性假瘤。非C-ROSE组60例患者术后明确病理诊断者45例(包括病灶EBUS-TBNA明确诊断11例、淋巴结EBUS-TBNA明确诊断34例)，术后未明确诊断的15例患者中有7例进一步有创操作后明确诊断(包括4例肺癌、1例肺结节病、1例淋巴结结核、1例胸腺细胞肿瘤)，6例患者随访影像学提示短期病灶明显增大伴肺外新发病灶故临床诊断为肺癌，2例患者抗感染治疗后随访影像学明显缩小故临床诊断为炎性假瘤；故非C-ROSE组60例患者包括54例肺癌、1例胸腺细胞肿瘤、1例肺结节病、2例淋巴结结核、2例炎性假瘤(表 4)。

**4:** 两组患者EBUS-TBNA术后病理诊断 Pathological diagnosis of two groups of patients by EBUS-TBNA

	Pathologic diagnosis	C-ROSE group	NO C-ROSE group
Definite diagnosis	Adenocarcinoma	35	12
	Squamous cell carcinoma	9	14
	Small cell lung cancer	15	14
	Non-small cell lung cancer	11	3
	Lung malignant tumor	1	1
	Other	1^#^	1^##^
No definite diagnosis		9	15
Total		81	60
^#^: lymphoma; ^##^: lymphoid tuberculosis.			

### 2.3 两组患者术后诊断率

将两组患者EBUS-TBNA术后的病理学结果与患者的最终诊断比较，计算两组患者病灶EBUS-TBNA、淋巴结EBUS-TBNA、EBUS-TBNA术后的诊断率(图 1)。病灶EBUS-TBNA术后两组诊断率无统计学差异，而C-ROSE组患者淋巴结EBUS-TBNA术后及整体EBUS-TBNA术后诊断率明显高于非C-ROSE组。

**1:**
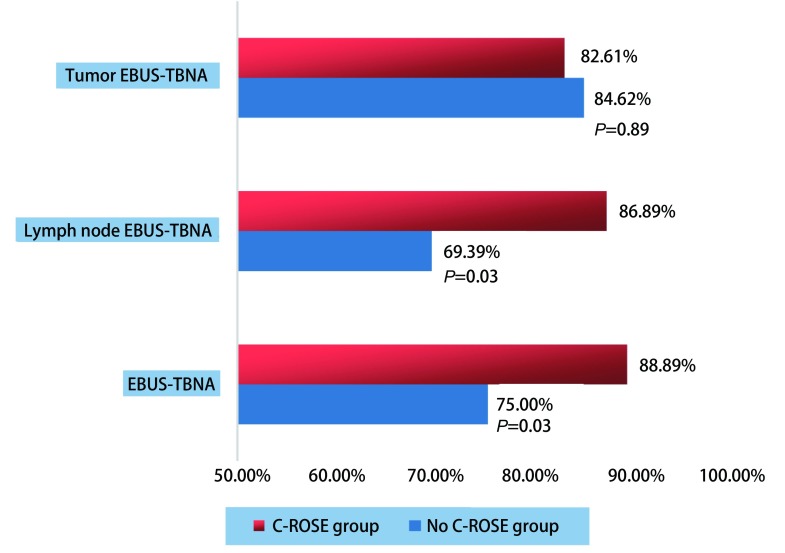
两组患者EBUS-TBNA术后诊断率 Postoperative diagnostic rate of two groups of patients by EBUS-TBNA

### 2.4 C-ROSE联合EBUS-TBNA取样诊断肺癌中的价值

C-ROSE联合EBUS-TBNA对肺癌诊断的敏感性、特异性、阳性预测值、阴性预测值分别为92.21%(71/77)、100.00%(4/4)、100.00%(71/71)、40.00%(4/10)。C-ROSE组中81例患者EBUS-TBNA术中70例快速现场细胞学找到肺癌细胞或者异型细胞，其中65例快速现场细胞学诊断与最终诊断一致；7例快速现场细胞学为假阴性，所有患者中无假阳性。

### 2.5 穿刺标本合格率分析

C-ROSE组及非C-ROSE组中分别有6例及1例术后未获得病理组织，仅获得抽吸液行细胞病理学检查。根据穿刺标本合格性判断标准，C-ROSE组及非C-ROSE组术后分别有1例及6例EBUS-TBNA穿刺标本不合格，两组穿刺合格率分别为98.77%和90.00%(*P*=0.02)，由此可见C-ROSE可以显著提高EBUS-TBNA穿刺标本合格率，减少二次操作或者创伤更大的进一步检查。

### 2.6 C-ROSE镜下细胞学形态分析

#### 2.6.1 C-ROSE镜下恶性肿瘤的形态学特点

分析总结C-ROSE组患者显微镜下所见肺癌细胞学形态的主要特点：①阅片后整体感觉肺癌细胞染色较深、体积较正常细胞明显增大且大小不等、形态不规则；②肺癌细胞的细胞成分较正常细胞明显增多，胞核大且多不规则，可以出现双核甚至多核，核/浆 > 1/2；③核仁增大且常呈现大小不等，可以有多个核仁，核仁的长径/核长径 > 1/4。

#### 2.6.2 不同类型的肺癌细胞C-ROSE镜下形态学特点

肺癌常见的三个病理类型为鳞状细胞癌、腺癌、小细胞未分化癌，不同类型的肺癌组织制片后显微镜下可见不同特点，本研究分析总结了不同病理类型肺癌行C-ROSE所见镜下形态学特征(图 2)。

**2:**
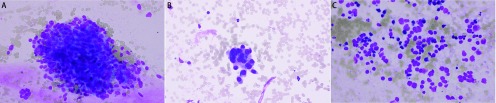
不同病理类型肺癌行C-ROSE所见镜下形态学特征(迪夫快速染色方法，×400)。A：鳞状细胞癌：大部分肿瘤细胞呈粘附紧密的大细胞簇、癌细胞及胞核形态畸形明显、背景示肿瘤性坏死明显(> 50%)；B：腺癌：部分肿瘤细胞呈粘附紧密的小/中等细胞簇、癌细胞较大且胞浆较丰富、核仁大而清楚；C：小细胞未分化癌：肿瘤细胞体积相对较小、核/浆比高甚至呈裸核、以单个散在分布的肿瘤细胞为主。 Classificatory morphologic characteristics of lung cancer with c-rose under microscope (Diff-Quik stain, ×400). A: Squamous carcinoma cells: Most of the tumor cells show large clusters of closely adhered cells, with obviously abnormal morphology and nucleus, and tumor necrosis is obvious in the background (> 50%); B: Adenocarcinoma cells: The cells show small clusters of tightly adhered cells, with large cancer cells, abundant cytoplasm, large and clear nucleolus; C: Small cell lung cancer: Tumor cells are relatively small, mainly with single scattered tumor cells, with a high nuclear/plasma ratio, even just bare nucleus.

### 2.7 并发症的比较

EBUS-TBNA术中并发症主要包括一过性低氧血症、一过性心律失常、穿刺部位出血，而其中最常见的并发症为出血，出血量由操作医师根据镜下表现估计，按文献所述^[11]^分为极少量出血(< 5 mL)、轻度出血(5 mL-20 mL)、中度出血(20 mL-100 mL)及重度出血(> 100 mL)；术后并发症主要包括发热、感染、痰中及咳嗽带血、纵隔气肿、气胸等。两组患者中均有较多例术中极少量出血和轻度出血，因其经过简单局部处理后出血即停止不影响后续操作，故不纳入本研究并发症统计范畴。C-ROSE组中有1例患者术中中度出血，为病灶EBUS-TBNA，共穿刺1针。非C-ROSE组中有5例患者术中中度出血，包括2例病灶EBUS-TBNA及3例淋巴结EBUS-TBNA，分别穿刺针数为2针、2针、4针、2针、1针；1例患者术后2 h内大咯血(一次性咯血150 mL)，为病灶EBUS-TBNA，共穿刺5针；1例患者术后皮下气肿明显，为淋巴结EBUS-TBNA，穿刺2针。两组患者中均无术中重度出血，术后气胸、纵隔气肿、发热、死亡患者。C-ROSE组的EBUS-TBNA并发症发生率明显低于非C-ROSE组(*P*=0.01)，分别为1.23%(1/81)和11.67%(7/60)。

### 2.8 联合免疫组化及基因检测技术

C-ROSE组共有33例(40.74%)行免疫组化协助诊治，共有13例(16.05%)行肺癌靶向药物基因检测，其中6例有突变基因并行靶向药物治疗；非C-ROSE组共有9例(15%)行免疫组化，共有10例(16.67%)行肺癌靶向药物基因检测，其中3例有突变基因并行靶向药物治疗。

## 3 讨论

肺癌在全球各个国家的发病率均较高，在我国更是发病率及死亡率都排第一的恶性肿瘤，现在中国已经成为了全世界肺癌人数最多的国家。因为早期无特异性临床表现、群众体检意识欠缺、经济欠发达等诸多因素，临床上很多肺癌患者初次就诊时则已经是中晚期，失去了手术治疗的机会、无法耐受创伤性大的操作，这就要求临床医师以最小创伤的方法、最快明确诊断并且尽早开始治疗。EBUS-TBNA是一种在呼吸内科应用十分成熟的内镜介入手术，对于影像学提示纵隔和(或)肺门病灶且疑诊肺癌的患者行EBUS-TBNA是一种行之有效的诊断手段。EBUS-TBNA的创伤小、耐受性较好，主要并发症为出血、低氧血症等，但可予以止血、吸氧或者置入喉罩连接呼吸机保护下手术等方式处理，本研究中C-ROSE组中接受EBUS-TBNA操作患者中最大年纪为83岁，相较于诊断的金标准纵隔镜，EBUS-TBNA检查具有较高的安全性、有效性及经济价值^[12]^。ROSE在欧美等现代化介入肺脏病诊疗中心几乎为“标配”^[8]^，其中C-ROSE应用最为广泛。国外有一些前瞻性研究认为EBUS-TBNA联合ROSE无明显受益^[11]^，但从减少并发症、提高“确切诊断”效率的角度，国外的一些多中心研究结果仍建议EBUS-TBNA应联用ROSE^[11]^。但国内呼吸介入水平及病理诊断水平较发达国家还有一定的差距，且国内因经济、人才等因素EBUS-TBNA及ROSE在临床工作中应用并不如国外应用广泛，也缺乏相关经验，国内有关EBUS-TBNA联合ROSE效益相关的研究也很少，专门针对肺癌的研究更少。

我们的研究表明EBUS-TBNA对肺癌的诊断率高，而术中联合C-ROSE后对肺癌的诊断率更是明显提高(75.00% *vs* 88.89%)，尤其是针对淋巴结EBUS-TBNA，这与Cardoso等^[6]^的研究结果一致。我们的研究还表明C-ROSE联合EBUS-TBNA可以显著提高穿刺标本合格率(90.00% *vs* 98.77%)，从而减少患者为明确诊断、指导治疗而行二次手术，避免进一步纵隔镜、胸腔镜、开胸手术等创伤更大的操作，有助于为患者减轻痛苦及经济负担。C-ROSE联合EBUS-TBNA对肺癌的诊断率高，但是仍有漏诊，考虑可能与操作者缺乏经验、淋巴结较小、标本量不足等有关系。我们的研究表明C-ROSE联合EBUS-TBNA还能显著减少并发症发生率，但两组患者的穿刺针数、穿刺深度无明显差异，分析原因如下：①在C-ROSE提示获得阳性标本时，我们并未单纯追求减少穿刺针数而直接停止穿刺，而是在保证安全的前提下个体化的根据患者C-ROSE评估标本满意度及临床需求决定是否再次穿刺。若是C-ROSE结果为阳性但恶性细胞比例不高或临床需要更多标本，且术中未出现并发症，则直接在原穿刺部位继续穿刺从而减少并发症；若是C-ROSE结果为阴性则改变穿刺部位。②两组穿刺针数无明显统计学差异可能与我科对于非C-ROSE组的患者平均穿刺针数较推荐的平均穿刺针数偏少有关，相关文献推荐EBUS-TBNA穿刺3针-5针^[13]^，而本研究中非C-ROSE组平均穿刺2.49针。但正因为C-ROSE组患者增加了穿刺针数，C-ROSE组的穿刺合格率及诊断率才显著高于非C-ROSE组，从而减少二次操作或者进一步更大创伤的操作，且在C-ROSE指导下并未增加并发症发生率。③可能因为本研究非前瞻性随机对照研究，部分混杂因素影响结果、纳入的样本量不足够大而使得结果有偏倚。

ROSE可以评估标本质量，在EBUS-TNBA中联用ROSE可以实时反馈给操作者有用的信息，决定是否需要再重复操作^[7]^，若评估确定获取足够靶标本则可以指导标本合理的分流送检并结束操作，除送检明确病理诊断，必要时可行免疫组织化学、聚合酶链反应、染色体荧光原位杂交、基因突变分析、特殊染色(如抗酸染色)等^[14]^，反之标本情况不满意，则操作继续，这有助于节省操作时间及医疗资源，减少患者创伤及并发症。

C-ROSE是EBUS-TBNA术中一种很好的辅助方法，但是我们千万不能过度限制穿刺针数，仅单纯追求组织病理学得到“阳性结果”。虽然更多的穿刺可能会延长操作时间、增加并发症发生率，但是如果不能明确诊断，不能获得足够标本行免疫组化或者靶向基因检测，那就要再次进行二次操作或者更大创伤的操作，付出的代价更大；而且EBUS-TBNA严重的并发症发生率不高，死亡人数极少^[15]^，而且我们研究表明C-ROSE联合EBUS-TBNA还可以降低并发症发生率。因此，临床医师可根据患者可能的诊治情况在操作时灵活处理，权衡利弊。另一方面，因为C-ROSE的结果和最后的病理诊断符合率很高^[16]^，我们的研究中C-ROSE组的81例患者EBUS-TBNA术中70例快速现场细胞学找到肺癌细胞或者异型细胞，其中65例快速现场细胞学诊断与最终诊断一致，故在EBUS-TBNA中联合ROSE可以快速给出倾向性诊断^[17]^，早期为进一步治疗做准备。

*Chest*的一项研究表明临床操作医师在经过3个月ROSE检查系统培训，其准确率可达80%，而专科的细胞病理学家为92%^[18]^，两者无显著统计学差异。但是鉴于并非国内全部内镜介入中心的工作人员都有机会接受细胞病理学方面的培训，故本研究总结了C-ROSE组肺癌患者显微镜下最常见的细胞形态学特点，有助于非病理专业的肺科从医人员识别肺癌细胞学特征、学习判读C-ROSE结果，从而明确诊断或缩窄鉴别诊断范围，结合临床信息判断病情。

ROSE最主要的临床意义包括以下几个方面：①提高微创取样标本质量，减少无谓穿刺，提高手术操作阳性率，减少并发症；②对疾病做出快速诊断，尽早为治疗做准备；③标本经评估充分后进行合理的分流送检，提高标本利用率并有利于疾病的诊断和治疗。ROSE技术所需设备简单，仅需一台显微镜、迪夫试剂，且ROSE技术仅需要少量细胞即可诊断，不损失取得的组织标本，故ROSE技术的引入是低投入、低风险的，长远看来，可以缩短检查时间、住院时间，减少相关费用。通过对我院141例EBUS-TBNA患者回顾性分析，认为C-ROSE在疑诊肺癌行EBUS-TBNA中应用可以提高穿刺成功率及诊断率、减少并发症，推荐在国内各呼吸内镜介入中心推广应用。
